# Biodistribution and serologic response in SARS-CoV-2 induced ARDS: A cohort study

**DOI:** 10.1371/journal.pone.0242917

**Published:** 2020-11-24

**Authors:** Tobias Schlesinger, Benedikt Weißbrich, Florian Wedekink, Quirin Notz, Johannes Herrmann, Manuel Krone, Magdalena Sitter, Benedikt Schmid, Markus Kredel, Jan Stumpner, Lars Dölken, Jörg Wischhusen, Peter Kranke, Patrick Meybohm, Christopher Lotz

**Affiliations:** 1 Department of Anesthesiology and Critical Care, University Hospital of Wuerzburg, Wuerzburg, Germany; 2 Institute for Virology and Immunobiology, University Hospital of Wuerzburg, Wuerzburg, Germany; 3 Institute of Obstetrics and Gynecology, University Hospital of Wuerzburg, Wuerzburg, Germany; 4 Institute for Hygiene and Microbiology, University of Wuerzburg, Wuerzburg, Germany; 5 Helmholtz Institute for RNA-based Infection Research (HIRI), Helmholtz-Center for Infection Research (HZI), Wuerzburg, Germany; National Institute for Infectious Diseases Lazzaro Spallanzani-IRCCS, ITALY

## Abstract

**Background:**

The viral load and tissue distribution of severe acute respiratory syndrome coronavirus 2 (SARS-CoV-2) remain important questions. The current study investigated SARS-CoV-2 viral load, biodistribution and anti-SARS-CoV-2 antibody formation in patients suffering from severe corona virus disease 2019 (COVID-19) induced acute respiratory distress syndrome (ARDS).

**Methods:**

This is a retrospective single-center study in 23 patients with COVID-19-induced ARDS. Data were collected within routine intensive care. SARS-CoV-2 viral load was assessed via reverse transcription quantitative polymerase chain reaction (RT-qPCR). Overall, 478 virology samples were taken. Anti-SARS-CoV-2-Spike-receptor binding domain (RBD) antibody detection of blood samples was performed with an enzyme-linked immunosorbent assay.

**Results:**

Most patients (91%) suffered from severe ARDS during ICU treatment with a 30-day mortality of 30%. None of the patients received antiviral treatment. Tracheal aspirates tested positive for SARS-CoV-2 in 100% of the cases, oropharyngeal swabs only in 77%. Blood samples were positive in 26% of the patients. No difference of viral load was found in tracheal or blood samples with regard to 30-day survival or disease severity. SARS-CoV-2 was never found in dialysate. Serologic testing revealed significantly lower concentrations of SARS-CoV-2 neutralizing IgM and IgA antibodies in survivors compared to non-survivors (p = 0.009).

**Conclusions:**

COVID-19 induced ARDS is accompanied by a high viral load of SARS-CoV-2 in tracheal aspirates, which remained detectable in the majority throughout intensive care treatment. Remarkably, SARS-CoV-2 RNA was never detected in dialysate even in patients with RNAemia. Viral load or the buildup of neutralizing antibodies was not associated with 30-day survival or disease severity.

## Introduction

The outbreak of the novel severe acute respiratory syndrome coronavirus 2 (SARS-CoV-2) was declared a pandemic by the WHO on March 11, 2020 [1]. As of September 01, 2020 more than 25 million people had been tested positive and more than 850000 people had died [2]. The proportion of patients requiring hospital treatment significantly increases with age [3]. Approximately 14–17% of hospitalized patients require intensive care due to an acute respiratory distress syndrome (ARDS) [4, 5].

In the wake of this pandemic, hospitals and intensive care units (ICU) are overwhelmed by the number of patients and health-care workers bear a high risk of SARS-CoV-2 infection themselves [6]. Containment strategies have focused on preventing airborne and droplet transmission, as viral loads are highest in respiratory secretions and seem to correlate with Corona virus disease 2019 (COVID-19) severity [7]. However, there is growing evidence for viral shedding within non-respiratory tissues. Recent studies reported SARS-CoV-2 RNA in blood (RNAemia) [7, 8], feces [7, 9, 10], urine [11], and peritoneal fluid [12, 13]. Some authors suggested a correlation between RNAemia and critical illness [14] whereas others found RNAemia in mild cases as well [11, 14]. Moreover, the serologic response to COVID-19 has recently received increasing attention. SARS-CoV-2 antibody levels increase between days eight to 14 which correlates with a slow but steady decline in viral load [15]. Prior studies further suggested that antibody titers are higher in severe clinical cases [16]. However, in many COVID-19 studies disease severity was largely undefined and the term *severe disease* included a wide range of clinical presentations.

The current retrospective study investigated SARS-CoV-2 viral load, biodistribution and anti-SARS-CoV-2 neutralizing antibody formation in critical ill patients suffering from moderate to severe COVID-19 induced ARDS during ICU treatment. We not only provide novel insights into COVID-19 in a high-risk population with over 50% extracorporeal membrane oxygenation (ECMO), but also important implications regarding the protection of ICU personnel from SARS-CoV-2.

## Methods

### Study design

This is a retrospective single-center cohort study adhering to the STROBE-Guidelines [17]. Approval by the Institutional Review Board of the University Hospital Wuerzburg, Germany and informed consent were waived due to sole retrospective chart review (63/20-kr; 25 March 2020).

### Patient selection

All patients (≥ 18 years) treated in the Intensive Care Unit of the Department of Anesthesiology and Critical Care at the University Hospital Wuerzburg (local ICU) between March 19, 2020 and May 06, 2020 with a confirmed diagnosis of COVID-19 were consecutively included in the study. Severity of ARDS was defined according to the Berlin definition [18].

All data were retrospectively extracted from the patient data management system between June 1^th^ - 7^th^, 2020 (COPRA6 RM1.0, COPRA System GmbH, Berlin, Germany), written records or communication with close relatives and general practitioners. Survival was defined as the survival 30 days after admission to the ICU. For patients discharged to general wards digital records were observed in order to determine the 30-day mortality.

### Sample collection

During ICU treatment oropharyngeal swabs, tracheobronchial aspirates, blood (serum or EDTA), urine, dialysate and anal swabs were longitudinally obtained to assess the viral load of COVID-19 patients. Sample collection was summarized for days 1 (ICU admission), 4, 7, 10 and 14. Samples that had been taken ± 1 day were assigned to the defined time points, respectively. Samples taken between days 20–28 were summarized as day 21. All specimens were analyzed during routine clinical care, specific treatment protocols were not defined. However, local standard protocols during the pandemic included a close monitoring of all potentially contaminated specimens. Overall, 478 virology samples were taken. Among those were 86 oropharyngeal swabs, 94 tracheobronchial aspirates, 96 blood samples, 85 urine samples, 33 dialysate samples, 83 anal swabs and 1 cerebrospinal fluid sample. Respiratory samples were obtained from all patients. Blood and urine samples were only available from 22, anal swabs from 21 and dialysate samples from 13 patients on continuous renal replacement therapy. In addition, one sample was obtained from cerebrospinal fluid at a single time point (day 3). Routine laboratory parameters included complete blood counts, markers of inflammation, immunoglobulin M, G and A levels as well as liver and renal function. Arterial blood gas samples were recorded multiple times per day.

### Reverse transcription quantitative polymerase chain reaction for SARS-CoV-2

Detection of SARS-CoV-2-RNA by Reverse transcription quantitative polymerase chain reaction (RT-qPCR) was carried out according to the recommendations of the World Health Organization [19] and the manufacturer’s instructions. Primers located in the SARS-CoV-2 E-gene as described by Corman et al. [20] and the test kit FTD SARS-CoV-2 (Siemens Healthineers, Erlangen, Germany) were used. Cycle thresholds (Ct) ≥40 were considered negative.

### Anti-SARS-CoV2-Spike-receptor binding domain (RBD) antibody detection

Serologic testing of blood samples was performed with an Enzyme-linked immunosorbent assay (ELISA) using the receptor-binding domain (RBD) of the SARS-CoV-2 Spike protein [21]. RBD was recombinantly expressed in Expi293F HEK cells (Expi293F HEK^TM^ cells, ThermoFisher Scientific#A14527, Lot# 1900411, authenticated by the provider) using the RBD_6His expression plasmid [22, 23]. The ELISA protocol was carried out as previously published [22, 23] with the following reagents: RBD was immobilized on NUNC (Nunc, Roskilde, Denmark) Maxisorp plates, blocking was done using 1x Roti-Block (Carl Roth; A151.1, Karlsruhe, Germany. Detection antibodies goat anti-human IgG [Thermo Fischer; 31410] and goat anti-human IgM [YO-Proteins; Ronninge, Sweden; ABIN301951] were diluted 1:10000, goat anti-human IgA [KPL International Limited, New Delhi, India, 5220–0360] was diluted 1:5000. For detection, ready-to-use s(hs)TMB (SDT-Reagents; sTMB, Baesweiler, Germany) was incubated for 10 minutes. Readout was performed on a Tecan sunrise (Tecan, Maennedorf, Switzerland) at 450 nm, corrected at 620 nm. An internal negative control pool was used to define the background signal. Since a reference antibody against RBD was not available at the time of the analyses, quantification was based on serum derived IgG (Sigma-Aldrich GmbH Munich, Germany; I2511), IgM (Sigma-Aldrich GmbH Munich, Germany; I8260) and IgA (Sigma-Aldrich; I4036) antibodies immobilized on NUNC Maxisorp plates. Standard curves were fitted using Origin (OriginLab Corporation, Northhampton, USA). R^2^ values for IgM: 0.99992, for IgG: 0.99964, and for IgA: 0.99965, respectively.

### Statistics

Data are reported as medians and interquartile range (IQR) (continuous data) or absolute numbers and percentages (categorical data). Normality of the data could not be assumed. We performed Fisher’s exact test for categorical variables and a Mann-Whitney-rank sum test for metric data. Two-sided statistical significance was set at p<0.05. Data were collected in Microsoft Excel. Statistical analyses were performed with SPSS 26 (IBM, Armonk, NY, USA) and Prism 8 (GraphPad Software, San Diego, CA, USA).

## Results

### Baseline characteristics and clinical course

A total of 23 patients met the inclusion criteria. Median age was 58 (49–69) years and the majority was male (65%). Baseline characteristics and co-morbidities are shown in **Table 1**. Two patients had a history of rheumatologic diseases and current medication with immunosuppressant (Prednisolone or Leflunomide), respectively.

**Table 1:** Demographic and baseline characteristics.

	Overall	Survivors	Non-Survivors	p-value
	(N = 23)	(n = 16)	(n = 7)	
Median age (IQR)–years	58 (49–69)	54 (48–69)	66 (56–69)	0.198
Sex–no. (%)				
Male	15 (65)	12 (75)	3 (43)	0.182
Female	8 (35)	4 (25)	4 (57)	
Median BMI (IQR)–kg/m^2^	29 (25–31)	28 (24–31)	31 (26–35)	0.193
Smoker–no. (%)				
Never	13 (57)	10 (62.5)	3 (43)	0.650
Former	10 (43)	6 (37.5)	4 (57)	
Active	0 (0)	0 (0)	0 (0)	
Co-morbidities–no. (%)				
Chronic obstructive pulmonary disease	9 (39)	7 (44)	2 (29)	0.657
Arterial hypertension	15 (65)	10 (63)	5 (71)	1.0
Coronary artery disease	3 (13)	1 (6)	2 (29)	0.209
Diabetes mellitus	5 (22)	3 (19)	2 (29)	0.621
Chronic renal insufficiency	2 (9)	1 (14)	1 (6)	0.526
Symptoms–no. (%)				
Dyspnea	12 (52)	9 (56)	3 (43)	0.667
Fever	22 (96)	16 (100)	6 (86)	0.304
Cough	18 (78)	12 (75)	6 (86)	1.0
Nausea	7 (30)	3 (19)	4 (57)	0.137
Diarrhea	7 (30)	3 (19)	4 (57)	0.137
Median duration (IQR)–days				
Symptom onset—hospital admission	6 (3–7)	6 (4–13)	5 (2–7)	0.281
Symptom onset–ICU admission	8 (5–10)	9 (5–14)	5 (5–9)	0.138
ARDS–no. (%)				
moderate	2 (9)	2 (12.5)	0 (0)	1.0
severe	21 (91)	14 (87.5)	7 (100)	
Median oxygenation index on admission (IQR)—P_a_O_2_/F_i_O_2_	140 (88–190)	134 (88–181)	164 (64–246)	0.769
Median worst oxygenation index during ICU treatment (IQR)—P_a_O_2_/F_i_O_2_	69 (54–97)	73 (59–97)	59 (49–82)	0.149
Duration of ICU treatment (local ICU)–days (IQR)	23 (13–41)	38 (17–43)	15 (8–20)	0.021
Duration of Mechanical ventilation–days (IQR)	20 (17–41)	39 (18–44)	18 (9–20)	0.028
Extracorporeal membrane oxygenation (ECMO)–no. (%)	15 (65)	10 (63)	5 (71)	1.0
Renal replacement therapy–no. (%)	17 (74)	10 (63)	7 (100)	0.124

Chronic obstructive pulmonary disease = chronic pulmonary obstructive disease or bronchial asthma; IQR = interquartile range; ICU = intensive care unit; BMI = body mass index; P_a_O_2_/F_i_O2 = arterial pressure of oxygen/fraction of inspired oxygen; Duration of Mechanical ventilation = time from intubation to extubation/decannulation or pressure support of or below 5 cm H_2_O for at least 24h; ECMO = extracorporeal membrane oxygenation; CVVHD = continuous venous-venous hemodialysis

**Fig 1:**
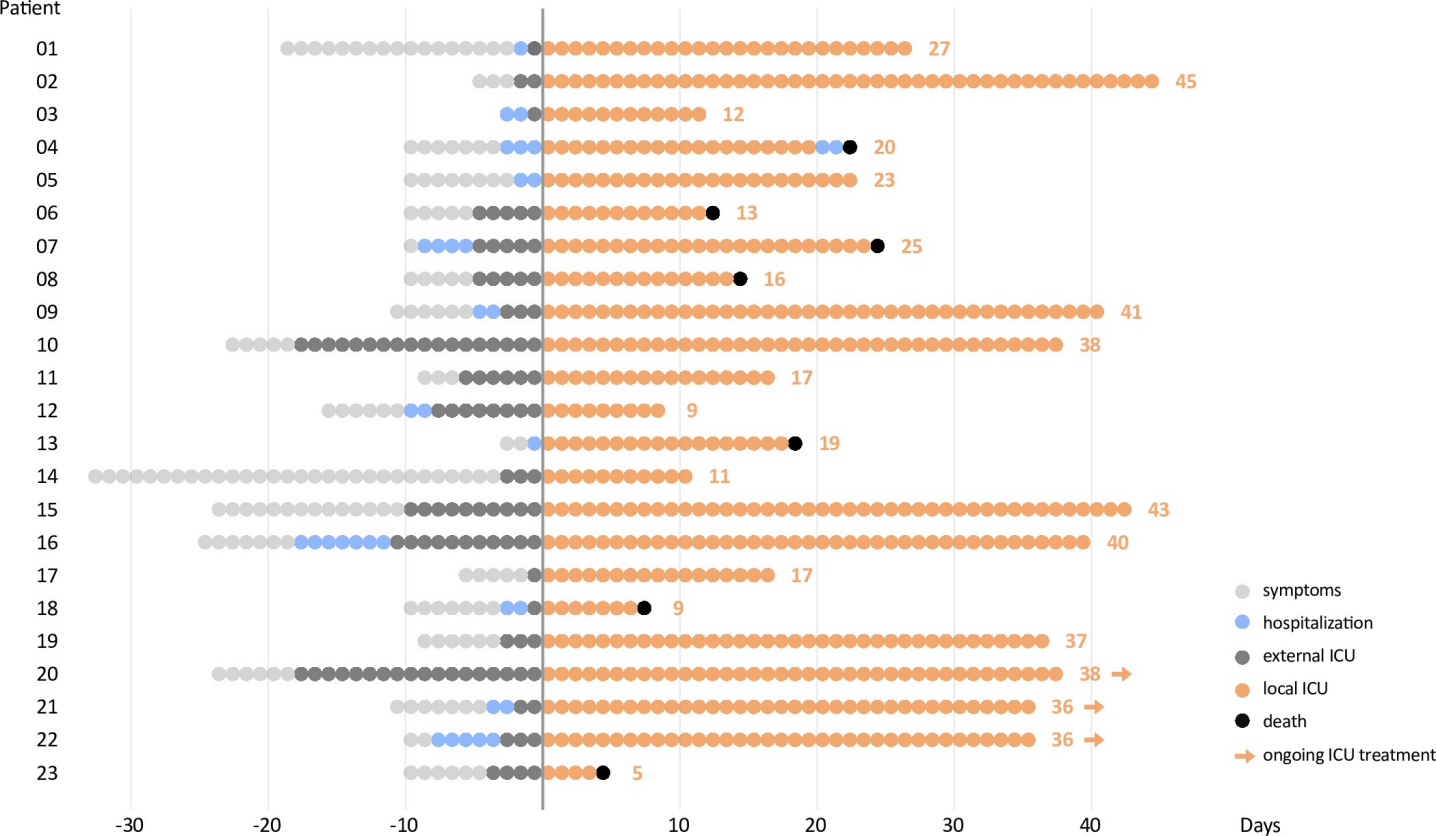
Clinical course of each COVID-19 ARDS patient within the study cohort. The figure starts with symptom onset and also displays hospital and ICU treatment prior transfer to the University Hospital of Wuerzburg (local ICU). Treatment at the ICU of the University Hospital Wuerzburg was defined as day 1. Patient 04 was transferred from the ICU to a general ward and died 3 days later.

All Patients had been transferred either from external ICUs (78%) or general wards (22%) due to worsening of pulmonary symptoms or increasing COVID-19-induced ARDS severity. Median duration (IQR) from symptom onset to hospital admission and ICU were 6 (3–7) and 8 (5–10) days, respectively (Fig 1).

Median P_a_O_2_/F_i_O_2_ oxygenation index (IQR) on admission was 140 mmHg (88–190). All 23 patients suffered from moderate (9%) or severe (91%) ARDS during ICU treatment with a median worst P_a_O_2_/F_i_O_2_ (IQR) of 69 (54–97). All patients were mechanically ventilated, 15 patients (65%) received extracorporeal membrane oxygenation (ECMO) and 17 patients (74%) were treated with continuous veno-venous hemodialysis (CVVHD). None of the patients received antiviral treatment. Mortality within 30 days after admission to the local ICU was 30%.

### SARS-CoV-2 viral load

**Fig 2:**
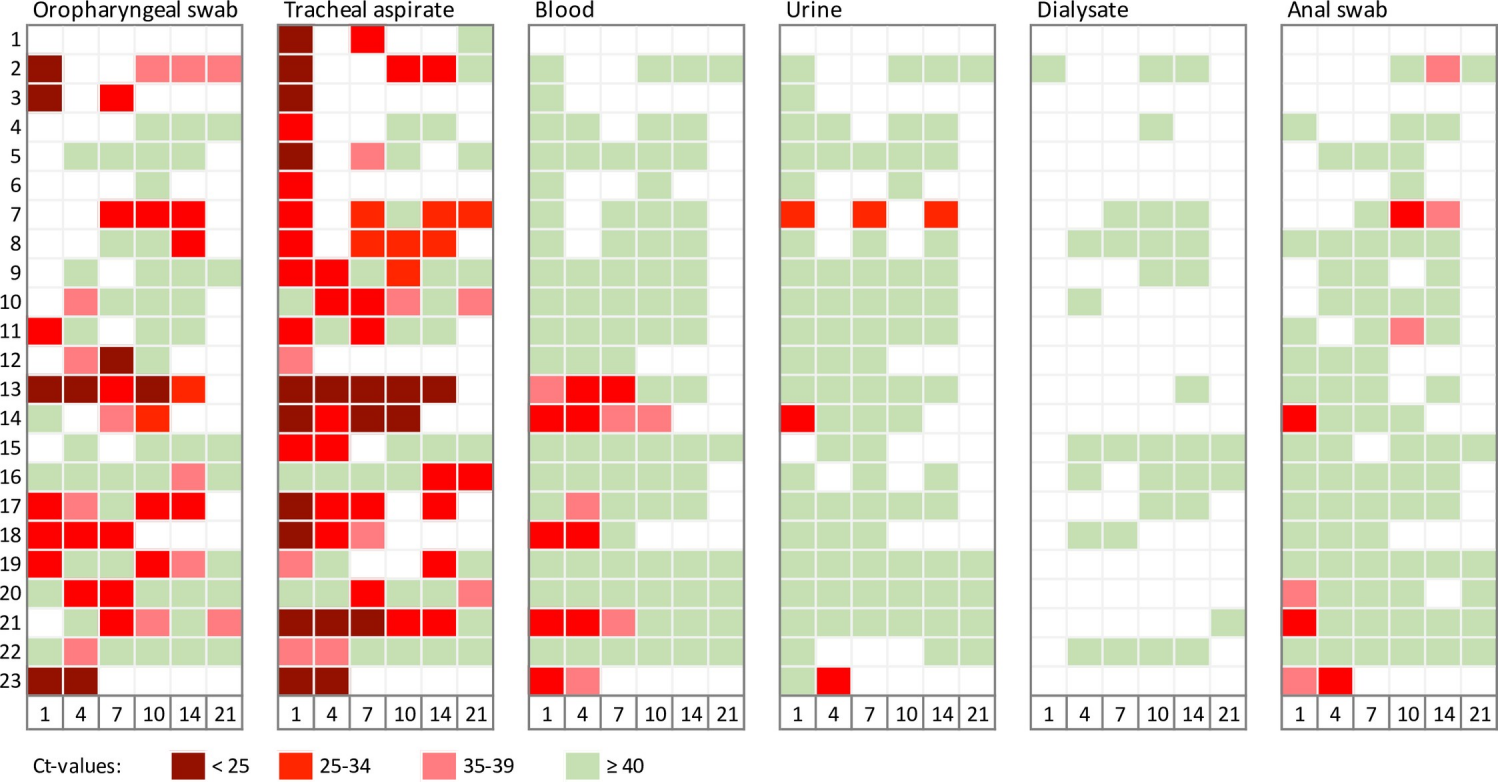
Viral load during treatment in local ICU (days 1, 4, 7, 10, 14, and 21 after ICU admission). Values of Cycle thresholds (Ct) are graduated. Lower values reflect higher viral loads. Values ≥ 40 were considered negative.

SARS-CoV-2 viral loads upon admission and during the course of ICU treatment are depicted in Fig 2. Tracheal aspirates tested positive for SARS-CoV-2 in all 23 cases (100%). Pharyngeal swabs tested positive for SARS-CoV-2 in 17 of 22 patients (77%). Simultaneous tracheal and oropharyngeal testing resulted in a negative oropharyngeal swab and a positive tracheal aspirate 15 times. The proportion of positive SARS-CoV-2 tests was much lower in non-respiratory samples: blood samples were positive in 26% of the patients. Urine tested positive in three (13%) and anal swabs in seven patients (30%). However, nearly half of the anal swabs provided positive test results for the first time after 10 days of ICU treatment. All samples of dialysate were negative. Cerebrospinal fluid was negative in one patient with concomitantly positive respiratory samples.

**Fig 3:**
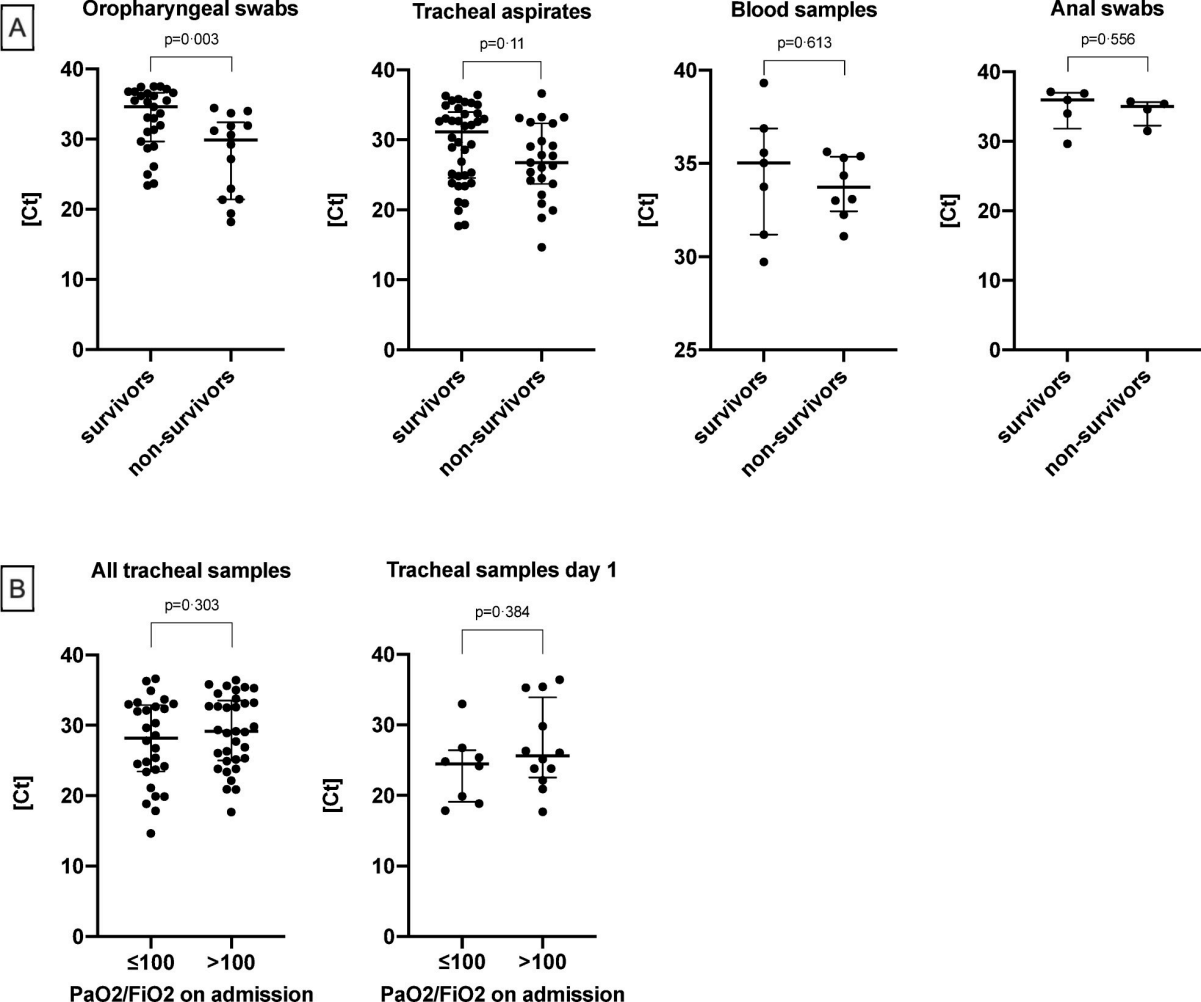
Median RT-qPCR cycles for SARS-CoV-2 RNA in all positive oropharyngeal swabs, tracheal aspirates, blood samples and anal swabs **(A)**. Values were compared between survivors and non-survivors. Ct-values of tracheal aspirates of all time-points as well as day 1 in the local ICU based on the oxygenation index on admission **(B).** Ct = cycle thresholds.

Cycle thresholds of positive samples differed between survivors and non-survivors: Median cycles of oropharyngeal swabs were 35 (30–37) in survivors and 30 (21–32) in non-survivors (p = 0.003). However, no significant difference was observed in tracheal swabs with 31 (25–34) in survivors and 27 (24–32) in non-survivors (p = 0.11). Median cycles (IQR) in blood samples were equal in survivors and non-survivors (p = 0.613). The same applied to median cycles of anal swabs (p = 0.556) (Fig 3A). Cycle thresholds of urine and dialysate samples are only graphically depicted (Fig 2) due to a low number or absence of positive results. Moreover, cycle thresholds in tracheal aspirates did not differ according to disease severity. No significant difference was found in samples from patients with moderate or severe ARDS (Fig 3B).

### Anti-SARS-CoV-2 neutralizing antibody formation

**Fig 4:**
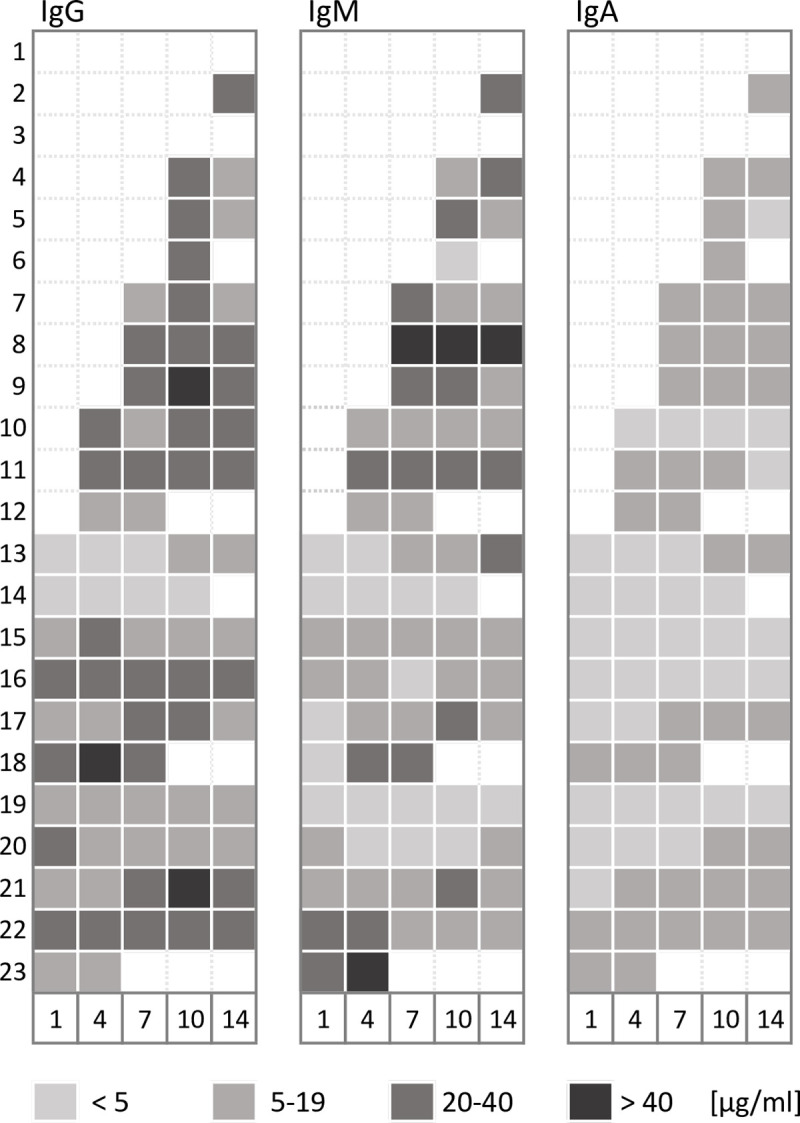
Levels and distribution of immunoglobulins (IgG, IgM, IgA) detected in blood samples (ELISA) on days 1–14. Serologic testing started later than virologic testing of blood samples, which explains discrepancies in the availability of results. White fields indicate unavailable results.

**Fig 5:**
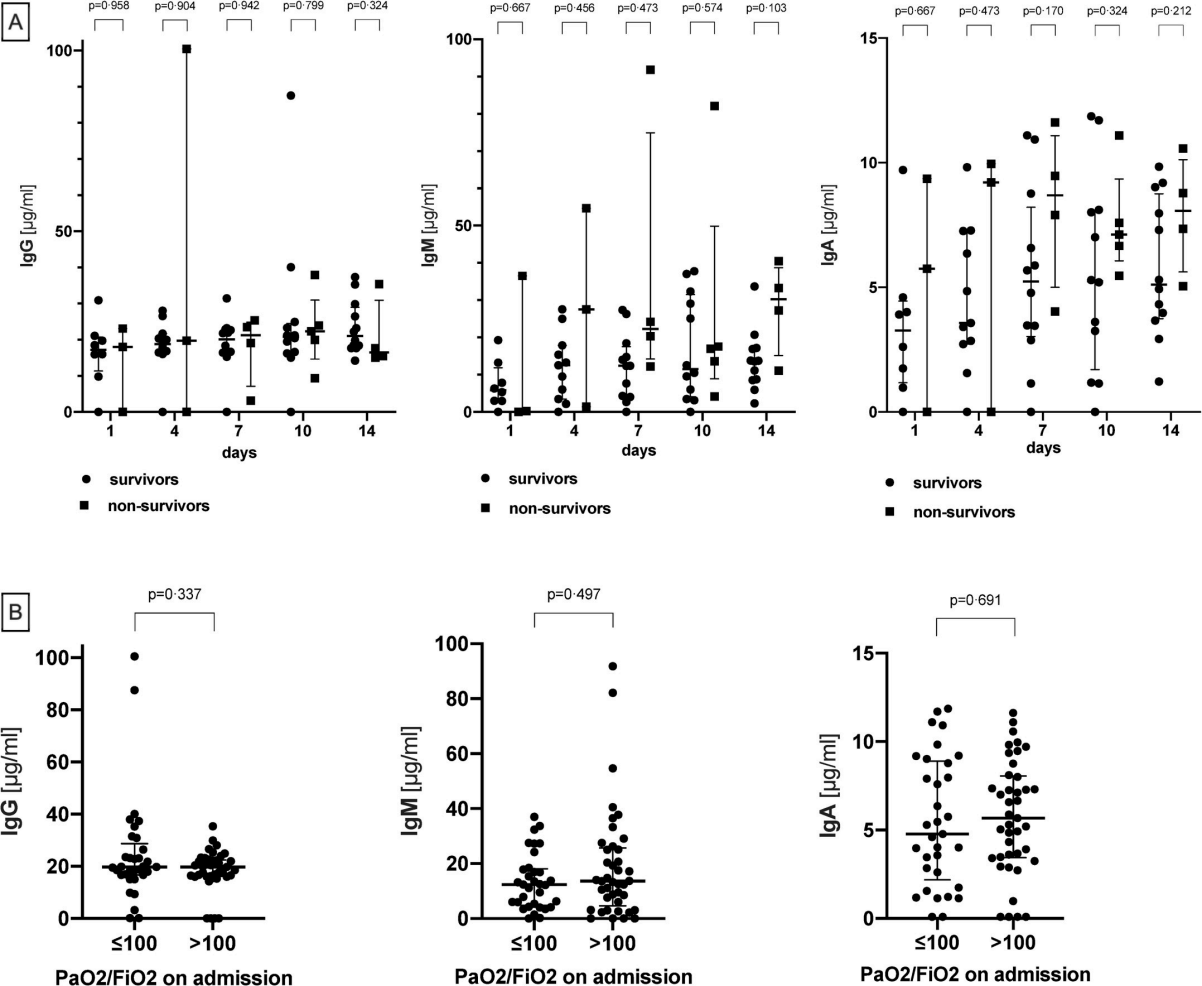
Comparisons of immunoglobulin serum concentrations (IgG, IgM and IgA) of survivors and non-survivors **(A)**. Comparisons of immunoglobulin serum concentrations (IgG, IgM and IgA) according to ARDS severity on ICU admission **(B)**.

Serologic tests were based on the detection of antibodies which bind to the receptor-binding domain of the SARS-CoV-2 Spike protein. In line with the biological function of this domain, an internal validation confirmed that such antibodies have neutralizing potential (data not shown). Serologic results of 21 patients revealed the presence of IgG, IgM and IgA neutralizing antibodies against SARS-CoV-2. All three immunoglobin classes were present in the same ten patients (43%) on ICU admission. In all but two patients (in which samples were not available) anti-SARS-CoV-2 IgG, IgM and IgA antibodies were detectable during the course of therapy (Fig 4). Median overall-concentrations of IgG were similar in survivors and non-survivors (20 [17–23] vs. 20 [15–24]; p = 0.99), whereas overall-concentrations of IgM and IgA were lower in survivors than in non-survivors (12 [4–18] vs. 20 [11–37]; p = 0.026 and 5 [3–7] vs. 8 [5–9]; p = 0.009). No difference in antibody concentrations was found when breaking down disease severity into moderate vs. severe ARDS on ICU admission (Fig 5).

**Fig 6:**
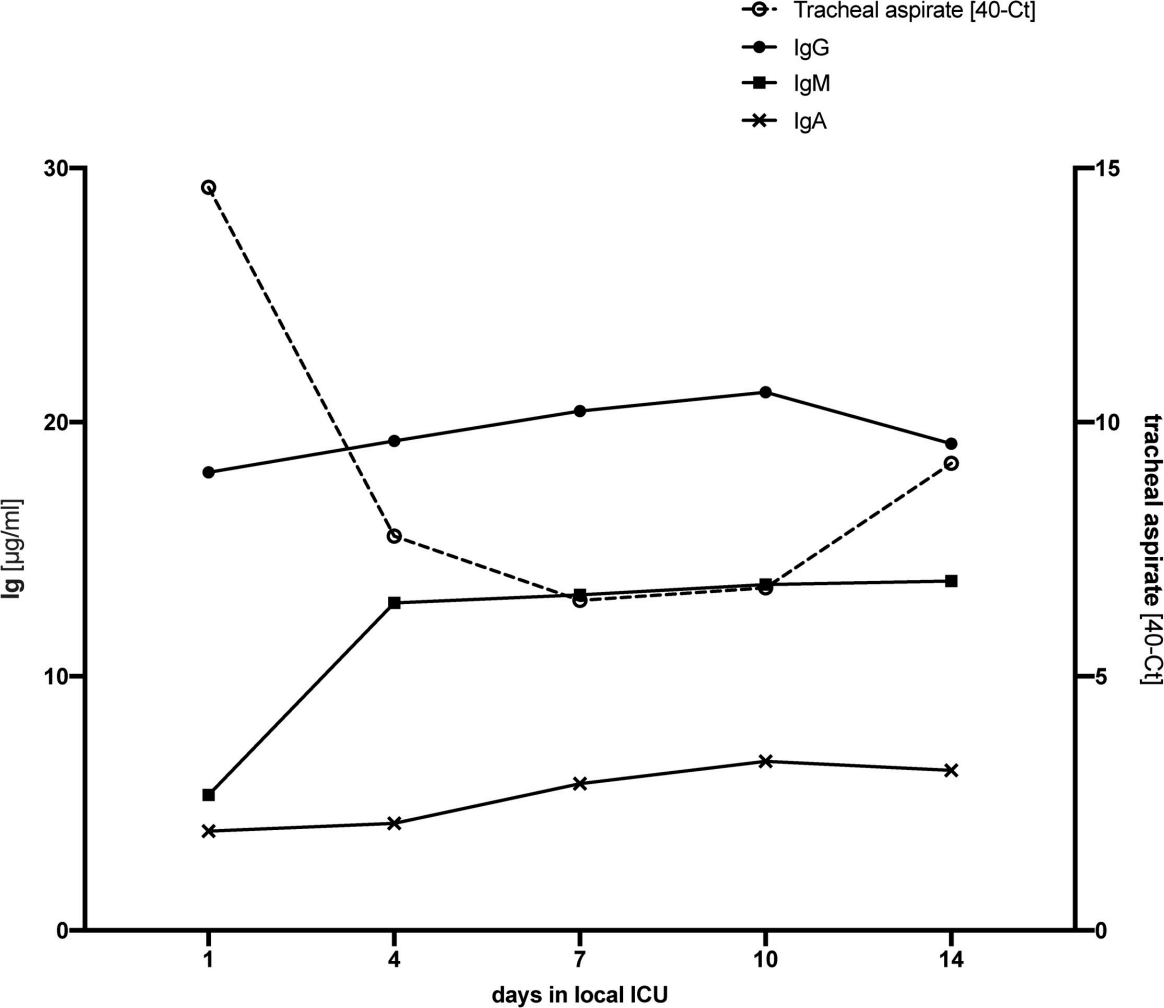
Immunoglobulin (Ig) G, M and A concentrations (medians) in relation to tracheal SARS-CoV-2 viral load over the course of the first 14 days of ICU therapy. SARS-CoV-2 loads in tracheal aspirate are displayed as medians of 40-Ct resulting from RT-qPCR.

The associations of tracheal viral load and neutralizing serum antibody concentrations are depicted in Fig 6. Tracheal viral load rapidly decreased within the first seven days of ICU treatment, whereas immunoglobin concentrations increased. As expected, IgM peaked earlier than IgG or IgA, nevertheless, on ICU admission IgG already was the most abundant immunoglobulin.

## Discussion

The infectiousness of SARS-CoV-2, viral load, clearance and severity of COVID-19 remain important and puzzling questions since the emergence of the pandemic. Our data indicate that severe COVID-19-induced ARDS is accompanied by a high viral load of SARS-CoV-2 in tracheal aspirates, which decreased over the course of ICU treatment. In the majority of patients (57%) tracheal virus RNA remained detectable throughout the course of ICU treatment. Neither viral load nor neutralizing antibody titers distinguished between moderate or severe ARDS. Moreover, both parameters were not different when comparing survivors and non-survivors. As such, our data do not allow a clear-cut distinction between these parameters and treatment or prognosis of COVID-19. We believe that this is an important finding itself and pinpoints towards pathophysiological mechanisms other than viral elimination contributing to disease severity and outcome. These mechanisms e.g. may include an impeded cytotoxic immune response [24] or microthrombosis due to platelet hyperactivation [25]. Oropharyngeal swabs expressed a similar pattern, however, results were less reliable and indicate that oropharyngeal swabs are prone to false negative results. Our data certainly emphasize that in patients receiving invasive mechanical ventilation a lower tracheal aspirate or bronchoalveolar lavage should be collected. In these patients, oropharyngeal swabs are likely not the most suitable specimens. Overall, detection of the highest viral load in respiratory samples confirms previous studies [7, 26].

Importantly, SARS-CoV-2 RNAemia was present in 26% of the patients, a finding contradictory to studies suggesting a direct correlation of RNAemia and severity of COVID-19. SARS-CoV2 RNAemia has been associated with more severe COVID-19, including a higher probability of ICU admission and mechanical ventilation. However, in these studies disease severity was either heterogenous [27] and ARDS severity remained undefined, respectively [28]. Only a minority of patients enrolled in these studies actually required intensive care, whereas our study only included critically ill ICU patients. Hence, although we did not find associations between viral load, RNAemia and survival it might be possible that RNAemia increases in-hospital mortality when considering all severities of COVID-19 [29]. Anal swabs and urine infrequently tested positive for SARS-CoV-2 RNA. Nevertheless, urine samples were positive in more cases than previously reported [9, 26, 30]. This could be an indication of a postulated renal tropism of SARS-CoV-2 [31]. The proportions of positive anal samples were in line with previous findings [9, 10], whereas a study from China reported no differences in blood and fecal viral load between mild and severe cases [7]. Positive anal swabs had previously been suggested as a potential indicator of critical disease, whereas the duration of virus RNA detection of the virus outlasted respiratory samples [32]. This potentially allows SARS-CoV2 transmission in other ways than respiratory droplets over an extended time-period. However, virus RNA detection within the digestive tract was not correlated with gastrointestinal symptoms [33]. It remains unclear why SARS-CoV2 RNA detection was delayed by up to ten days after ICU admission in some of our patients. However, impaired gastrointestinal mobility and delayed defecation is common in critically ill patients and may explain these findings.

Remarkably, SARS-CoV-2 RNA was never detected in dialysate from renal replacement therapy even in patients with RNAemia, indicating that the virus does not cross the dialysis membrane. Hence, dialysis fluid can probably be safely handed and disposed by the ICU personnel. This is an important finding, as a high proportion of severely ill ICU patients require renal replacement therapy, which is in turn associated with handling a high volume of potentially infectious fluid.

Antibodies against SARS-CoV-2 can be detected approximately 7–14 days after symptom onset [34]. The SARS-CoV2 spike protein recognizes and binds to the host receptor angiotensin-converting enzyme (ACE) 2, subsequently mediating viral cell membrane fusion. It is pivotal for SARS-CoV2 infection and a potential therapeutic target including vaccine development. Antibodies against the spike protein are immunodominant and can neutralize SARS-CoV-2 [35]. However, antibody kinetics, acquired immunity, duration of protection, as well as the potential occurrence of mutations disrupting neutralizability remain unknown. This also applies to its relation to disease severity. Patients may be protected from future SARS-CoV2 infection by their acquired immunity, suffer from moderate COVID-19 or even experience increased severity of infection due to immunopathogenesis. The latter may be relevant to both first and second exposure, particularly in an ICU study population [36].

Considering a median of 8 days between symptom onset and ICU admission in our patients, the presence of these antibodies in the majority of patients is expected. In two patients, however, antibody formation was not observed. Both of these patients had been on long-term immunosuppressive medication prior to COVID-19. One of those patients survived ICU treatment. Three classes of antibodies, i.e. IgM, IgA and IgG, could be found at the time of ICU admission and during the course of ICU therapy. IgG antibody titers did not differ between survivors and non-survivors, while IgM and IgA were significantly higher in non-survivors. Studies have found high virus specific antibody titers correlating with enhanced virus elimination [16, 37]. The observed difference might reflect different stages of disease or differences in the interplay between the humoral and cytotoxic immune defense. The observed lower anti-SARS-CoV-2-Spike-RBD IgM levels in survivors may indicate clinical recovery as IgM is the antibody class which predominates in the early stages of an immune response and is the first to be produced. However, other data imply that higher titers might be associated with more severe disease [16, 37]. This finding was also present in patients dying from SARS-Co-V1 [38]. Of course, all patients were critically ill in our cohort, and there was no general association between survival and antibody titers. Interestingly, antibody titers plateaued between days 7 to 14, which likely indicates that antibody formation was fully developed at that time point. In some cases, viral RNA remained detectable even after seroconversion. A lack of complete viral clearance was previously reported and is confirmed by the present results [39]. However, the detection of SARS-CoV-2 RNA alone is not sufficient to diagnose active disease and always need to be interpreted within the clinical context.

In this cohort of ICU patients, most patients (91%) suffered from severe ARDS during the course of therapy with a survival of 70%. Moreover, 65% were supported with ECMO and 74% with renal replacement therapy. None of the patients received antiviral treatment. Median age, prevalence of co-morbidities and duration between symptom onset and ICU admission were in line with previous studies [7, 40]. Hospitalization and ICU treatment were significantly longer [41] and compared to a previous investigation our cohort exhibited higher rates of invasive ventilation, need for ECMO therapy, as well as a higher mortality [30].

When interpreting the results of the current study it is important to emphasize the severity of disease in our patient population. Our data represent ICU patients surviving the worst clinical course of COVID-19 while receiving maximum ICU care. The majority of studies which have assessed SARS-CoV-2 viral loads to date, mainly reported mild cases [7, 9, 42, 43]. Moreover, viral load, virus elimination and neutralizing antibody formation are only some components of the complex COVID-19 pathophysiology. Further limitations include incomplete data in a few patients due to the retrospective design and collection of data within standard care. Generally, all findings regarding virus detection must be interpreted with caution since viral load as assessed via RT-qPCR may also reflect differences in sampling. Furthermore, RT-qPCR can only detect virus RNA but not infective particles. The latter would require cell culture, which is time-consuming and not sensitive enough [44] to be of use in acute clinical care. Being aware that the detection of virus RNA is not proof of intact virus or infectivity, RT-qPCR still remains the standard of care in SARS-CoV-2 diagnostics and the best surrogate for viral load [5]. Moreover, based on an early decision to closely monitor biodistribution and the serologic response, we are confident that the reported results in general represent the SARS-CoV-2 biodistribution and viral loads in the described cohort of severely ill COVID-19 patients.

## Supporting information

S1 File (DOCX)

## Abbreviations

SARS-CoV-2: severe acute respiratory syndrome coronavirus 2
Covid-19: corona virus disease 2019
ICU: intensive care unit
ARDS: acute respiratory distress syndrome
RBD: receptor binding domain
ECMO: extracorporeal membrane oxygenation
RT-qPCR: Reverse transcription quantitative polymerase chain reaction
ELISA: enzyme linked immunosorbent assay
Ct: cycle thresholds
RNAemia: ribonucleid acid detected in blood
