# Supplementary material for: Biodistribution and serologic response in SARS-CoV-2 induced ARDS: A cohort study
Source: PLoS One. 2020 Nov 24;15(11):e0242917. doi: 10.1371/journal.pone.0242917 (PMC7685466; doi:10.1371/journal.pone.0242917)
Supplement: S1 File — (DOCX) [file pone.0242917.s001.docx]

## Anti-SARS-CoV-2 Neutralizing Antibody Formation and Testing

Sensitivity, specificity (i.e. discrimination between SARS-CoV-2 and other coronaviruses), and neutralizing potential critically depend on selection of the target antigen. The ELISA used in our study was established early during the pandemic. Initial analyses performed in April 2020 revealed no cross-reactivity with antibodies in historical serum cohorts from 2017 (n=20). Cross-comparison against a neutralization assay (similar to [1]) showed complete concordance in our test cohort (n=11). Subsequently, groups at UCSF evaluated the RBD-specific ELISA and 12 commercial tests [2] and found the RBD-ELISA to be the most specific (>99% based on 108 historical samples) and among the most sensitive tests available. Thereafter, an adapted peer-reviewed version of the test protocol was published in Nature Medicine [3]. A most recent publication [4] estimates that a 100% specific ELISA against the SARS-CoV-2 Spike protein receptor binding domain could show sensitivities of 95% for IgG, 90% for IgA, and 81% for IgM for detecting infected individuals between 15 and 28 days after symptom onset. The good performance of the test is likely to be explained by the immunodominance and specificity of the novel receptor-binding domain of SARS-CoV-2 [5]. Moreover, antibodies binding to the receptor-binding domain of the SARS-CoV-2 Spike protein are likely to interfere with viral entry. This was confirmed by Iyer et al. [4] who found that IgG antibodies to SARS-CoV-2 RBD were strongly correlated with anti-S neutralizing antibody titers. Thus, the choice of the antigenic bait and the data obtained when setting up the ELISA test (which still represents the state-of-the-art) render it likely that the detected antibodies also possessed neutralizing activity. Still, as this could not be tested on the available samples, this has to remain an (albeit reasonable) assumption.

**References**

1. Lei C, Qian K, Li T, Zhang S, Fu W, Ding M, et al. Neutralization of SARS-CoV-2 spike pseudotyped virus by recombinant ACE2-Ig. Nature Communications. 2020;11(1):2070.

2. Whitman JD, Hiatt J, Mowery CT, Shy BR, Yu R, Yamamoto TN, et al. Test performance evaluation of SARS-CoV-2 serological assays. medRxiv. 2020:2020.04.25.20074856.

3. Amanat F, Stadlbauer D, Strohmeier S, Nguyen THO, Chromikova V, McMahon M, et al. A serological assay to detect SARS-CoV-2 seroconversion in humans. Nat Med. 2020;26(7):1033-6.

4. Iyer AS, Jones FK, Nodoushani A, Kelly M, Becker M, Slater D, et al. Persistence and decay of human antibody responses to the receptor binding domain of SARS-CoV-2 spike protein in COVID-19 patients. Science Immunology. 2020;5(52):eabe0367.

5. Premkumar L, Segovia-Chumbez B, Jadi R, Martinez DR, Raut R, Markmann A, et al. The receptor binding domain of the viral spike protein is an immunodominant and highly specific target of antibodies in SARS-CoV-2 patients. Sci Immunol. 2020;5(48).
